# The Thyroid Swing: A Patient's Journey From Hypothyroidism to Hyperthyroidism

**DOI:** 10.7759/cureus.52598

**Published:** 2024-01-19

**Authors:** Haifa Alnahdi

**Affiliations:** 1 Department of Internal Medicine, Endocrinology and Metabolism, Faculty of Medicine, King Abdulaziz University, Jeddah, SAU

**Keywords:** hypothyroidism, antibodies, levothyroxine, hyperthyroidism, hashimoto’s, graves´disease

## Abstract

The flip from hyperthyroidism (HT), as observed in Graves' disease (GD) cases, to hypothyroidism associated with Hashimoto's disease (HD) has been widely recognized and documented. However, the converse is infrequently observed. The observed phenomenon is likely attributed to the major role played by thyroid-stimulating hormone receptor antibodies (TRAbs) in this alternating disease pattern and a substantial reduction in thyroid tissue mass that could be stimulated by these antibodies. Therefore, here we present a case of one middle-aged individual experiencing this transitional switch from hypothyroidism to GD. A 42-year-old male patient diagnosed with HD-related hypothyroidism for two years has remained stable with levothyroxine medication since diagnosis. However, his symptoms of thyrotoxicosis, such as anxiety, weight loss, and palpitations, had appeared during his follow-up time at the clinic. Physical examination revealed mild exophthalmos, which confirmed HT by thyroid function test results. The possibility of levothyroxine-induced HT was initially considered, although the observed symptoms failed to ameliorate despite the reduction and discontinuation of levothyroxine. The diagnosis of GD was confirmed after subsequent examination, and a follow-up was carried out. This particular case study underscores a distinctive correlation that carries substantial implications for the diagnosis and treatment of GD. The consideration of this shift is warranted in cases where HT continues to exist despite the reduction or cessation of levothyroxine treatment. The diagnosis is established through antibody titers and a radioiodine uptake scan. Treatment choice is based on the illness's stage and the patient's and care provider's preferences. In the initial phase, anti-thyroid medications may be employed. It is imperative to conduct regular follow-ups on these patients due to the potential transient nature of this clinical condition.

## Introduction

Autoimmune thyroid diseases primarily encompass Hashimoto's disease (HD) and Graves' disease (GD), with the transition from GD to HD-associated hypothyroidism being common. However, the reverse transition is rare, potentially due to the reduced thyroid mass that can be stimulated by antibodies, making such a shift unusual [1]. Both HD and GD are leading causes of hypothyroidism and hyperthyroidism (HT), respectively [2,3]. GD-related HT often becomes hypothyroidism post-radioactive iodine or surgical treatment or, less commonly, shifts to HD-induced hypothyroidism when the immune system changes from stimulating to inhibiting the thyroid [4].

Conversely, the transition from HD-related hypothyroidism to HT is not common and is rarely reported [4]. In cases where patients treated for HD with levothyroxine show thyrotoxicosis symptoms, it usually points to an excessive medication dose. Still, the potential progression to GD warrants investigation. This report details a patient's unusual shift from hypothyroidism to GD two years after initial diagnosis, alongside a thorough literature review exploring this rare change's mechanism, characteristics, and treatment strategies.

In Saudi Arabia, the prevalence of thyroid dysfunction varies. A study indicated an 18.7% incidence of hypothyroidism [5], while a single-center retrospective study in Jeddah noted hypothyroidism in 29.1% of 3872 participants [6]. Research in the Kingdom primarily focuses on primary care and lacks broader general population insights. Moreover, comprehensive studies on the etiology and immunology of thyroid dysfunction are scant. Another study in Hail corroborated the higher prevalence of thyroid dysfunction in females, a trend consistent with global observations [7].

## Case presentation

A 42-year-old male, initially diagnosed with subclinical hypothyroidism while being assessed for male infertility in 2019, was put on a daily 100 mcg levothyroxine regimen. This treatment was effective, maintaining a euthyroid state. The patient also had hypertension, peptic ulcer disease, and gout, which were treated with amlodipine, esomeprazole, and allopurinol, respectively. The patient, a non-smoker and non-drinker, worked at a military installation and had a family history of hypothyroidism.

In 2022, after two years of stable thyroid function on levothyroxine, the patient presented to our facility with symptoms indicative of HT, such as palpitations and weight loss, despite the medication being halved to 50 mcg. Levothyroxine was discontinued when a thyroid function test showed undetectable thyroid-stimulating hormone (TSH) levels. The patient's clinical exam revealed tachycardia, a body mass index (BMI) of 34.4, and a diffuse nontender goiter, but no eye or skin abnormalities.

Subsequent investigations confirmed the suspicion of GD. Laboratory results showed a TSH level of <0.01 mIU/L, with elevated free T4 at 24.4 pmol/L (Table 1). A positive anti-TSH receptor antibody and thyroid peroxidase antibody were present. Hematologic and biochemical parameters, including WBC, hemoglobin, platelets, and liver and kidney function tests, remained within normal ranges (Table 2).

**Table 1:** TSH, free T3, and free T4 trend during follow-up. TSH: Thyroid-stimulating hormone

Test	Normal Range	Jan 2019	Jan 2022	Feb 2022	Apr 2022	Aug 2022	Dec 2022
TSH	0.3 – 4.2 IU/L	8	<0.01	0.004	0.24	4.1	4
Free T4	12 – 22 Pmol/L	16	24	34	12.6	12.18	12.42
Free T3	2.8 –7 Pmol/L	N/A	N/A	12	3.59	4.28	4.15

**Table 2:** Autoantibody results during the clinical follow-up. TPO Ab: Thyroid peroxidase antibody, TRAB: TSH receptor antibody, TSI: Thyroid-stimulating immunoglobulin

Antibody Test	Normal Range	Patient Results
TPO Ab	0–5.6 IU/ml	865
Antithyroglobulin Ab	0–4.1 IU/mL	2.3
TRAB	<1.75 IU/L	23.9
TSI	<140%	180%

**Figure 1:**
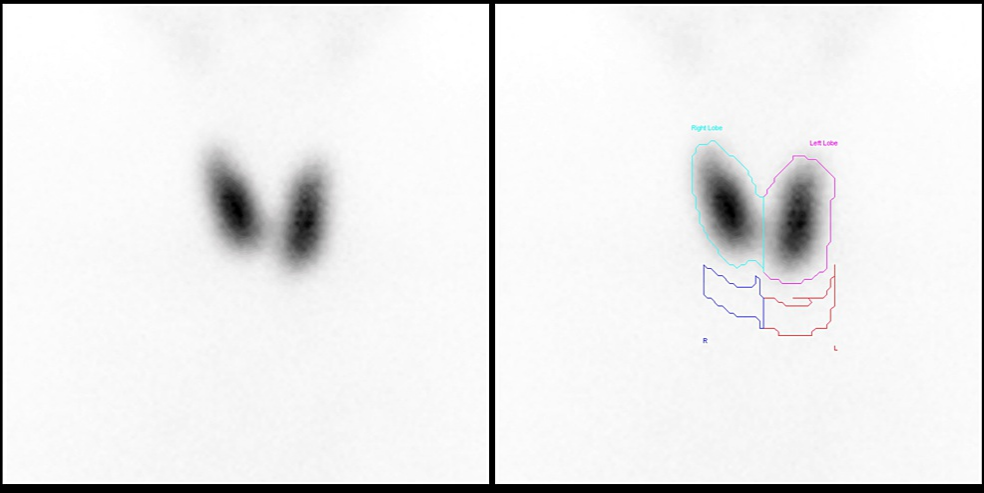
Results of the thyroid nuclear scan using technetium 99m. The thyroid shows a uniform and widespread absorption of substances without any signs of a toxic nodule or thyroiditis. These occurrences may potentially be Indicative of the initial stages of Graves' illness.

The TC-99 thyroid scintigraphy reveals a notable diffuse homogeneous uptake of the thyroid gland accompanied by a decrease in background activity, consistent with GD (Figure 1). 

Given the underlying autoimmunity and the potential for fluctuation between two spectra, the patient could pursue a definitive treatment, including radioactive iodine or thyroidectomy. However, the patient expressed a preference for initiating anti-thyroidal medication. The patient was administered suppressive medication consisting of a daily oral dose of 20 mg of carbimazole and propranolol. A follow-up conducted three months later revealed that restoring thyroid function to normal levels improved the patient's clinical condition.

## Discussion

GD is widely recognized as the primary etiology of HT. This condition is classified as an autoimmune disorder, exhibiting the concurrent presence of ophthalmopathy, dermopathy, and goiter. The TSH receptor antibody (TRAB) serves as a distinctive marker for GD, with high levels of sensitivity and specificity [3]. In certain instances, the presence of TPO antibodies may also be identified. Nevertheless, the presence of thyroid peroxidase antibodies (TPO Ab) is typically observed in the majority of instances of Hypothyroidism, accounting for over 90% of diagnoses [1]. GD and HD exhibit similarities in their pathophysiology related to autoimmune pathogenesis. While rarely, the coexistence of these situations is possible [8].

Hypothyroidism can occur in up to 20% of individuals who have previously received treatment with anti-thyroid medications for GD, persisting even after the initial treatment [9,10] and extended durations [10]. The presence of symptoms associated with excessive thyroid hormone activity in individuals with an underactive thyroid typically indicates excessive treatment, which can be addressed by adjusting the thyroid hormone dosage. The transition from hypothyroidism related to HD to GD is rare. The observed phenomenon is believed to be attributed to the significant reduction in thyroid volume, which can interact with thyroid-stimulating hormone receptor antibodies (TSHR Ab) [10]. While rarely, this transition carries noteworthy therapeutic implications and, as a result, necessitates prompt clinical identification. 

A scoping assessment of the existing literature was conducted to investigate this seemingly uncommon phenomenon's mechanism, prevalence, and characteristics. We encountered a new study by Gonzalez-Aguilera et al. [4]. In the retrospective assessment of a cohort including 2000 patients who sought medical care at their clinic, it was seen that 24 individuals (1% of the total sample) had a transition from HD to GD. The study found that the median duration of the transformation was 18 months. The majority of cases (22 out of 24) involved females. Only four instances exhibited ocular abnormalities; all cases were initially classified as medication-induced HT. The investigation and diagnosis of GD were prompted by the persistence of symptoms, as observed in our specific instance. According to Gonzalez-Aguilera et al., it has been hypothesized that the pathophysiology of this shift involves a transition like TSH receptor antibodies from blocking (TBAb) to stimulating (TSAb), or it may be attributed to an initial occurrence of severe thyroid damage leading to hypothyroidism, which then transitions to HT as a result of the stimulation of thyroid hormones, during the recovery of the thyroid gland. In our case study, the initial explanation appears more plausible, given that the individual got GD after two years, more than 18 months, as observed in Gonzalez's study [4].

Takasu et al. documented a substantial cohort of adult patients, consisting of eight individuals, representing the second biggest pool of subjects in sample size. Furthermore, the study included a lengthy follow-up period to assess the long-term outcomes of these patients [11]. A proposal was put out to categorize this particular group of patients into three distinct groups, considering the presence of TSAb and the duration of symptoms. There are two distinct groups to consider in this context. The first group pertains to cases of temporary HT related to GD following a period of hypothyroidism. The second group, on the other hand, involves instances of persistent HT connected to GD after a period of hypothyroidism. Lastly, the third cohort consists of individuals who exhibit persistent hypothyroidism accompanied by positive TSAb levels. In the series under investigation, most patients (71%, n = 5/7) exhibited a temporary condition, hence obviating the requirement for targeted anti-thyroid medication. In two instances, the symptoms endured, requiring administering anti-thyroid treatment (2 out of 7). One instance exhibited the presence of positive thyroid-stimulating antibodies (TSAb), although they did not manifest HT. Our literature review also yielded supplementary individual case reports [11-17]. In 2012, Kamath et al. proposed the involvement of an immunological shift as a potential mechanism for the manifestation of Graves’ disease, concluding that the current mechanism is speculative at this stage [18].

TPO antibodies remained positive in our case study, as shown by the detection of thyroid antibodies. It should be noted that TRAB antibody levels were not previously assessed. The patient exhibited continued good health during the follow-up period while receiving anti-thyroid treatment. Given the potential occurrence of temporary HT, which appears to be prevalent in our patient, we must regularly observe the patient for any indications or manifestations of hypothyroidism. The possible shift from Hashimoto's to Graves' suggested a need for vigilant follow-up for potential hypothyroid reversion and antibody activity changes affecting thyroid function [19,20].

## Conclusions

When hypothyroid patients show thyrotoxicosis, excluding levothyroxine overdose, it is crucial to consider GD. Diagnosis requires detailed history, physical exams, antibody titers, and radioiodine scans. Our case highlights the possibility of a shift from Hashimoto's to Graves', suggesting a need for vigilant follow-up for potential hypothyroid reversion and changes in the antibody activity affecting thyroid function.
